# Exosomal miR-17-3p Alleviates Programmed Necrosis in Cardiac Ischemia/Reperfusion Injury by Regulating TIMP3 Expression

**DOI:** 10.1155/2022/2785113

**Published:** 2022-01-25

**Authors:** Zhuyuan Liu, Didi Zhu, Fuchao Yu, Mingming Yang, Dan Huang, Zhenjun Ji, Wenbin Lu, Genshan Ma

**Affiliations:** Department of Cardiology, Zhongda Hospital, Southeast University, Hunan Road, Nanjing, 210009 Jiangsu, China

## Abstract

**Objective:**

Myocardial ischemia/reperfusion (I/R) injury can aggravate myocardial injury. Programmed necrosis plays a crucial role in this injury. However, the role of exosomal miRNAs in myocardial I/R injury remains unclear. Therefore, this study is aimed at exploring the function and mechanism of exosomal miR-17-3p in myocardial I/R injury.

**Methods:**

The myocardial I/R injury animal model was established in C57BL/6 mice. Exosomes were identified using transmission electron microscopy (TEM), nanoparticle tracking analysis (NTA), and Western blotting. Programmed necrosis was detected by PI staining. Heart function and myocardial infarct size were evaluated using echocardiography and triphenyl tetrazolium chloride (TTC) staining, respectively. Histopathological changes were visualized by hematoxylin and eosin (H&E) and Masson staining. The regulation of TIMP3 expression by miR-17-3p was verified using a dual-luciferase reporter assay. Lactate dehydrogenase (LDH) and tumor necrosis factor-*α* (TNF-*α*) levels were measured by enzyme-linked immunosorbent assays (ELISA). TIMP3 expression was measured by quantitative reverse transcription-polymerase chain reaction (qRT-PCR) and Western blotting.

**Results:**

We demonstrated that miR-17-3p was significantly downregulated in peripheral blood exosomes after cardiac I/R injury. Further analysis indicated that exosomal miR-17-3p attenuated H_2_O_2_-induced programmed necrosis in cardiomyocytes in vitro. Moreover, TIMP3 was a target for miR-17-3p. TIMP3 affected H_2_O_2_-induced programmed necrosis in cardiomyocytes. This effect was modulated by miR-17-3p in vitro. Furthermore, exosomal miR-17-3p greatly alleviated cardiac I/R injury in vivo.

**Conclusions:**

The present study demonstrated that exosomal miR-17-3p alleviated the programmed necrosis associated with cardiac I/R injury by regulating TIMP3 expression. These findings could represent a potential treatment for I/R injury.

## 1. Introduction

Myocardial ischemia/reperfusion (I/R) injury is a common pathophysiological phenomenon in the clinic [1]. Reperfusion is an effective treatment for ischemic heart disease, such as acute myocardial infarction (AMI). However, reperfusion is often performed after the blood flow in myocardial tissue is interrupted for a certain period of time and then reestablished. Under those conditions, reperfusion fails to restore normal heart function and aggravates its dysfunction and structural damage, leading to irreversible damage that could affect the prognosis of patients with myocardial infarction [2, 3]. Therefore, clarifying the mechanism of myocardial I/R injury has important clinical significance for reducing or eliminating this injury.

The pathogenesis of myocardial I/R injury is complex, and oxygen free radicals, calcium overload, and inflammatory mediators are recognized mechanisms [1, 4]. MicroRNAs (miRNAs) are small noncoding RNAs with a length of about 22 nucleotides that are widely present in various organisms and regulate growth, development, and apoptosis. miRNAs (e.g., microRNA-374a, miR-204-3p, and microRNA-193b) are involved in various diseases and myocardial I/R injury [5–7]. In addition, an increasing number of studies have found that exosomes and exosomal miRNA play a crucial role in myocardial I/R injury. For example, mesenchymal stem cell-derived exosomal miRNA-181a and miR-182 attenuate myocardial I/R injury by influencing the inflammatory response and macrophage polarization, respectively [8, 9]. Exosomes derived from the coronary serum of patients with myocardial infarction promote angiogenesis through the miRNA-143/IGF-IR pathway [10].

Programmed cell necrosis is a new type of cell death that is different from apoptosis and traditional necrosis [11]. An increasing number of studies have demonstrated that programmed necrosis plays an important role in myocardial I/R injury [12]. Several studies also found that miRNA can protect against myocardial I/R injury by regulating this process. For example, miRNA-325-3p protects the heart after myocardial infarction by inhibiting RIPK3 and programmed necrosis in mice [13]. In addition, miRNA-103 and miRNA-107 regulate programmed necrosis and myocardial I/R injury by targeting FADD [14]. miR-17-3p contributes to exercise-induced cardiac growth and protects against myocardial I/R injury [15]. miR-17-3p plays a crucial role in different diseases by regulating various kinds of biological functions. Rosuvastatin attenuates myocardial I/R injury by mediating autophagy by upregulating miR-17-3p [16]. Recently, Yuan et al. demonstrated that H/R injury in H9C2 cardiomyocytes is reduced by modulating inflammatory signaling regulated by miR-17-3p [17]. Moreover, we found that increasing miR-17-3p levels from serum extracellular vesicles promotes H9C2 proliferation [18]. However, the function and mechanism of exosomal miR-17-3p in programmed necrosis during myocardial I/R injury remain unclear. This study is aimed at exploring the mechanism of exosomal miR-17-3p in programmed necrosis during myocardial I/R injury in vitro and in vivo, providing a potential therapeutic target for cardiac I/R injury.

## 2. Materials and Methods

### 2.1. Exosome Isolation and Characterization

Exosomes were isolated using the Total Exosome Isolation Kit (4484450, Invitrogen, USA) according to the manufacturer's instructions. The size distribution and concentration of exosomes were measured by nanoparticle tracking analysis (NTA) using a ZetaView instrument (Particle Metrix). For transmission electron microscopy (TEM), exosomes were sedimented by ultracentrifugation (Beckman TL-100) at 200,000 × g for 20 h through a sucrose gradient and fixed in 3% (*w*/*v*) glutaraldehyde and 2% paraformaldehyde in cacodylate buffer (pH 7.3). The fixed exosomes were applied to a continuous carbon grid and negatively stained with 2% uranyl acetate. The samples were examined with a Philips CM10 electron microscope. The exosomal markers (CD63, CD9, and TSG101) were also detected by Western blot.

### 2.2. Cell Culture, Transfection, and H_2_O_2_ Treatment

Rat primary cardiomyocytes were isolated and cultured in Dulbecco's modified Eagle's medium (Gibco, Thermo Fisher Scientific Inc.) containing 10% fetal bovine serum (Gibco, USA) at 37°C in an incubator with 95% air and 5% CO_2_. Cells were used in experiments at 70% confluency. H_2_O_2_ treatment to cause the ischemic reperfusion (I/R) injury was performed as previously described [14, 19, 20]. Briefly, the cardiomyocytes were treated with 500 *μ*M H_2_O_2_ (Sigma, USA), 10 *μ*g/mL exosomes, or both 500 *μ*M H_2_O_2_ and 10 *μ*g/mL exosomes for 24 h. For transfection, the cardiomyocytes were transfected using Lipofectamine® 3000 reagent (Invitrogen; Thermo Fisher Scientific Inc.), according to the manufacturer's protocol. The cells were harvested 48 h after transfection for subsequent experiments. The sequences of the miR-17-3p inhibitor, small interfering RNAs (siRNAs) targeting TIMP3, and the negative control siRNAs (si-NC) were designed and synthesized by KeyGEN BioTECH. The sequences were as follows: miR-17-3p inhibitor 5′-UUCUCCGAACGUGUCACGUTT-3; Si TIMP3 sense, 5′-GGUCUACACUAUUAAGCAAAU-3′; Si TIMP3 antisense, 5′-UUGCUUAAUAGUGUAGACCAG-3.

### 2.3. Cell Necrosis Assays

Cell necrosis was detected by PI staining. Briefly, cardiomyocytes were washed three times with PBS after H_2_O_2_ treatment. The cells were stained with 1.5 *μ*M PI on ice for 5 min and then fixed with 4% PFA on ice for 30 min after washing with PBS three times. The cardiomyocytes were counterstained with DAPI and examined with a Nikon Eclipse Ti-S fluorescence microscope. Image analysis was performed with Image-Pro Plus software. The percentage of necrotic cell death was calculated by the total number of PI-positive nuclei/total DAPI-stained nuclei.

### 2.4. Quantitative Reverse Transcription-PCR (qRT-PCR)

Total RNA was extracted from the cardiomyocytes and tissues using TRIzol® reagent (Biosharp BS259A). The quality and concentration of the RNA were measured using a NanoDrop, and 1 *μ*g RNA was reverse transcribed into cDNA using the PrimeScript RT Reagent Kit with gDNA Eraser (Vazyme MR101-01/02) for mRNA and Super RT Kit (Bioteke Biotechnology Co. Ltd.) for miRNA, according to the manufacturers' protocols. qRT-PCR was performed using SYBR Premix Ex Taq II (Vazyme Q711-02) and the manufacturer's protocol. cDNA was amplified using the following thermocycling conditions: 94°C for 10 min (94°C for 20 s, 60°C for 20 s, and 72°C for 20 s) for 40 cycles; relative gene expression was analyzed using the 2^−ΔΔ*Ct*^ method. *β*-Actin and U6 were used as the internal standards for mRNA and miRNA, respectively. The primers were synthesized by Tsingke Biotechnology Co. Ltd., and the primer sequences are listed in Table 1.

### 2.5. Western Blot Analysis

Total protein was extracted from cultured cells or tissues using RIPA buffer (Thermo Fisher Scientific). Protein concentrations were determined using the BCA assay (Biosharp BL521A). Equal amounts of protein (20 *μ*g) were separated on SDS-PAGE gels and transferred to polyvinylidene difluoride (PVDF) membranes (Millipore). The membranes were blocked with 5% nonfat milk in 10% Tris-buffered saline Tween 20 (TBST) at room temperature for 1 h. The membranes were then incubated with CD9 (1 : 2000, ab92726, Abcam), CD63 (1 : 2000, ab134045, Abcam), TSG101 (1 : 2000, ab125011, Abcam), TIMP3 (1 : 1000, ab276134, Abcam), ZNF28 (1 : 1000, PA5-48229, Invitrogen), FAM83F (1 : 1000, ab272651, Abcam), and Neurog1 (1 : 500, sc-100332, Santa Cruz) primary antibodies overnight at 4°C. The membranes were incubated with goat anti-rabbit peroxidase-conjugated secondary antibodies (Biosharp BL003A) at room temperature for 2 h. The signal was detected using the ECL system (Millipore, Massachusetts, USA), according to the manufacturer's instructions. The relative protein expression was analyzed using ImageJ software and presented as the density ratio between the target protein and the *β*-actin internal control.

### 2.6. Bioinformatics Analysis and Dual-Luciferase Reporter Assay

TargetScan (http://www.targetscan.org/vert_72/) was used to identify the potential miR-17-3p binding sequences in TIMP3. Wild-type and mutant 3′UTR of TIMP3 sequences were cloned into the pmirGLO vector downstream of the luciferase reporter gene. The reporter constructs were cotransfected into cardiomyocytes with a miR-17-3p mimic or mimic NC using Lipofectamine 3000 according to the manufacturer's instructions. Luciferase activity was measured 48 h after transfection using the Dual-Luciferase Reporter Assay System (Promega, Madison, WI) following the manufacturer's instructions.

### 2.7. Myocardial I/R Model in C57BL6 Mice

Ten-week-old C57BL/6 mice were purchased from the Comparative Medicine Center of Yangzhou University and housed in a specific-pathogen-free facility with a 12 light-dark cycle under 21 ± 2°C with 55% air humidity. The myocardial I/R model was established as previously described [20]. Briefly, mice were anesthetized with 1.5–2.0% isoflurane and ventilated with room air using a rodent ventilator. A thoracotomy was performed at the fourth intercostal space to expose the heart and left anterior descending coronary artery (LAD). For the negative control, the LAD was not ligated. For functional studies, C57BL/6 mice were intravenously injected with 5 mg/mL exosomes (150 *μ*L) or the 100 nM miR-17-3p inhibitor (100 *μ*L) one day before myocardial I/R injury. All animals were anesthetized by isoflurane inhalation (1.5–2%) and then euthanized by cervical dislocation. The myocardial infarct size was detected with Evans blue dye and 2,3,5-triphenyl-2H-tetrazolium chloride (TTC) staining. The area of infarction and total area of the transverse section were measured using NIH ImageJ software. The infarct size (%) = infarct size/total area of transverse section × 100%. Echocardiography was performed on the rats in each group using a MyLab 30 CV ultrasound system (Esaote, S.P.A, Genoa, Italy) with 10 MHz linear ultrasonic transducers. The ejection fraction (EF (%)), fraction shortening (FS (%)), and heart rate were measured. The left ventricular end-diastolic diameter (LVEDd) and left ventricular end-systolic diameter (LVEDs) were also measured. LVEF (%) = [(left ventricular end-diastolic volume − left ventricular end-systolic volume)/left ventricular end-diastolic volume] × 100%. Lactate dehydrogenase (LDH) and TNF-*α* were determined using the LDH assay kit (Nanjing Jiancheng Bioengineering Institute A020-2-2) and TNF-*α* enzyme-linked immunosorbent assay (Elabscience E-EL-M0049C), according to the manufacturers' protocols. Absorbance was detected at a wavelength of 450 nm using an ultramicro microporous plate spectrophotometer (BioTek Instruments Inc.). All animal experiments were performed in accordance with the Guide for the Care and Use of Laboratory Animal by International Committees. The protocol was approved by the Institutional Animal Care and Use Committee of Southeast University.

### 2.8. Hematoxylin-Eosin and Masson Staining

Cardiac tissues were fixed with 4% paraformaldehyde for 24 h. Paraffin-embedded tissues were cut into 6 *μ*m sections and dried at 45°C. For H&E staining, the tissues were stained with hematoxylin for 3 min and then placed in 1% hydrochloric acid in ethanol for 2 sec. The sections were then stained with eosin for 3 min, dehydrated with gradient alcohol, permeabilized with xylene, sealed with resinene, and dried for 72 h. For Masson staining, the tissue sections were dewaxed with xylene (three times for 5 min each) and soaked in an alcohol gradient. The sections were stained using the Masson kit (Solarbio G1345). Changes in inflammatory infiltration and myocardial fibrosis were observed using light microscopy (Olympus Mountain, Tokyo, Japan), and images were analyzed using Image-Pro Plus software.

### 2.9. Statistical Analysis

Data were analyzed using SPSS 20.0 (IBM) and presented as the mean ± SD with at least three repeats. Differences between two groups and multiple groups were determined using the Student's *t*-test and one-way ANOVA with Tukey's test, respectively. *P* < 0.05 was considered statistically significant.

## 3. Results

### 3.1. Exosomal miR-17-3p Was Significantly Downregulated in the Peripheral Blood of Patients with Cardiac I/R Injury

**Figure 1:**
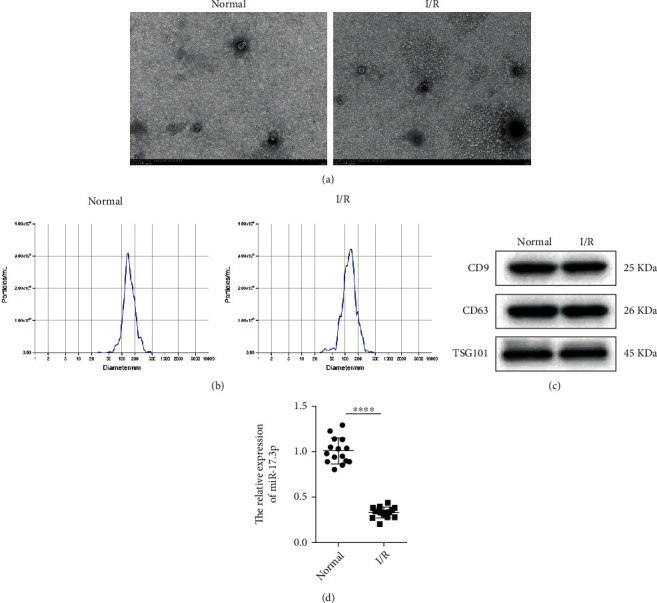
miR-17-3p was significantly downregulated in peripheral blood exosomes from patients with cardiac I/R injury. (a) The morphology of the peripheral blood exosomes was detected by TEM (*n* = 3, bar = 100 *μ*m). (b) The concentration and diameters of exosome particles were determined using the NTA assay (*n* = 3). (c) The expression of exosomal markers (CD63, CD9, and TSG101) was evaluated by Western blotting (*n* = 3). (d) The expression of exosomal miR-17-3p in the peripheral blood was measured by qRT-PCR (*n* = 15). U6 was used as a control. Data are presented as the mean ± SD; ^∗∗∗∗^*P* < 0.001, I/R injury vs. control.

To understand the function of exosomal miR-17-3p, exosomes were isolated from the peripheral blood of patients with cardiac I/R injury or healthy individuals. TEM analysis showed that the isolated exosomes had typical exosomal morphology, including a cup-shaped canonical appearance with a double-layer membrane (Figure 1(a)). Nanoparticle tracking analysis (NTA) showed that most particles were 100–200 nm in diameter, with a peak at 150 nm (Figure 1(b)). In addition, Western blotting revealed that the exosomal markers CD63, CD9, and TSG101 were highly expressed (Figure 1(c)), indicating that exosomes were successfully isolated from the peripheral blood. To understand the function of miR-17-3p, miR-17-3p expression levels in the exosomes from the different samples were measured by qRT-PCR. As shown in Figure 1(d), miR-17-3p expression was significantly decreased in the peripheral blood exosomes from patients with cardiac I/R injury compared to healthy individuals. These results demonstrated that exosomal miR-17-3p in peripheral blood might play a crucial role in cardiac I/R injury.

### 3.2. Exosomal miR-17-3p Attenuated H_2_O_2_-Induced Programmed Necrosis in Primary Cardiomyocytes In Vitro

**Figure 2:**
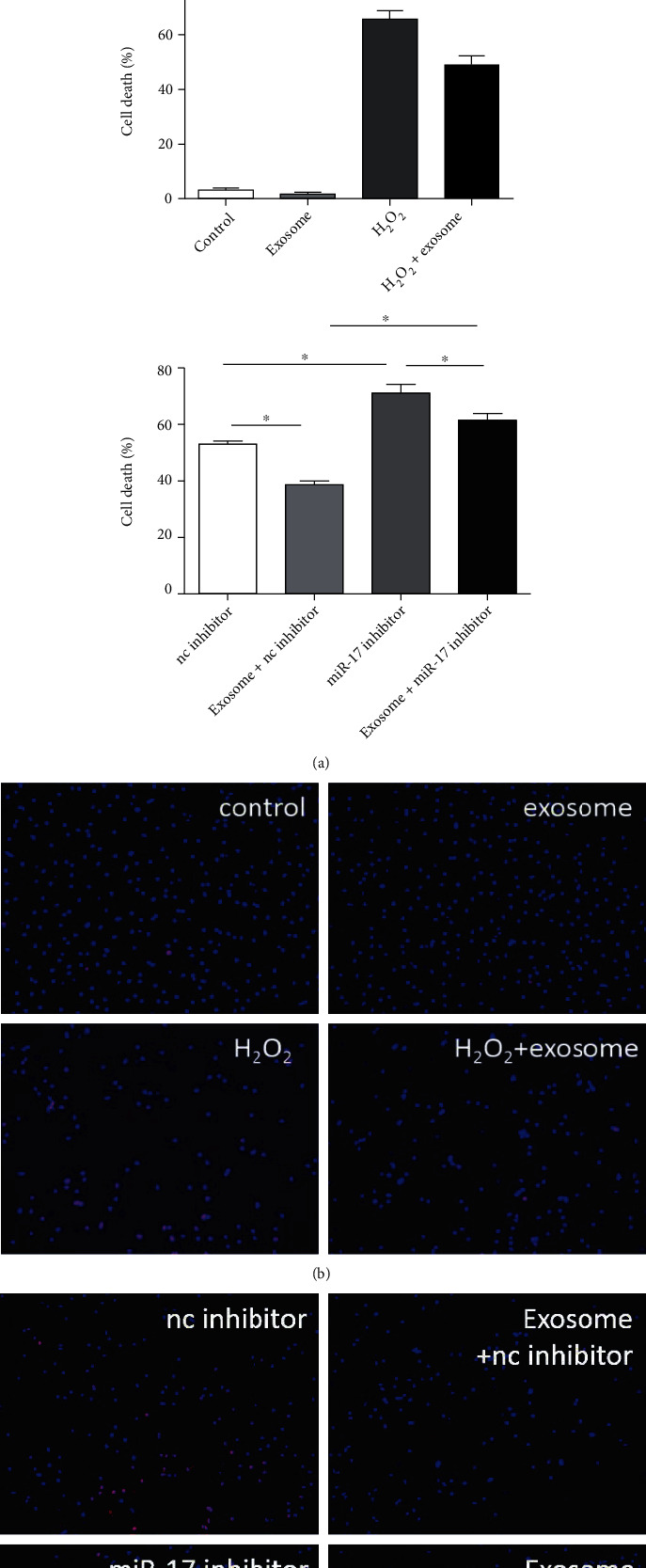
Exosomal miR-17-3p attenuated H_2_O_2_-induced programmed necrosis in primary cardiomyocytes in vitro. (a) miR-17-3p expression was measured by qRT-PCR in primary cardiomyocytes treated with peripheral blood exosomes (*n* = 3) using U6 as the control gene. (b, c) Necrotic cell death was assessed using PI staining following treatment with peripheral blood exosomes or the miR-17-3p inhibitor (*n* = 3). The representative images show the PI-positive cells (bar = 100 *μ*m). Data are presented as the mean ± SD; ^∗^*P* < 0.05; ^∗∗∗∗^*P* < 0.001.

The effect of exosomal miR-17-3p on H_2_O_2_-induced programmed necrosis in primary cardiomyocytes was investigated by treating the cells with exosomes. As shown in Figure 2(a), miR-17-3p expression was significantly elevated following the treatment of control or H_2_O_2_-induced primary cardiomyocytes with peripheral blood exosomes. miR-17-3p expression was much lower in H_2_O_2_-induced primary cardiomyocytes than in the control cells. Moreover, the number of primary cardiomyocytes undergoing programmed necrosis was dramatically increased following treatment with H_2_O_2_; however, treatment with exosomes significantly reduced the number of H_2_O_2_-induced necrotic primary cardiomyocytes (Figure 2(b)). Furthermore, inhibition of miR-17-3p expression in H_2_O_2_-induced primary cardiomyocytes by transfection with a miR-17-3p inhibitor significantly increased the number of necrotic primary cardiomyocytes (inhibitor vs. NC), which was partially rescued by treatment with exosomes (Figure 2(c)). These results suggested that exosomal miR-17-3p attenuated H_2_O_2_-induced programmed necrosis of primary cardiomyocytes in vitro.

### 3.3. TIMP3 Is a Direct Target for miR-17-3p

**Figure 3:**
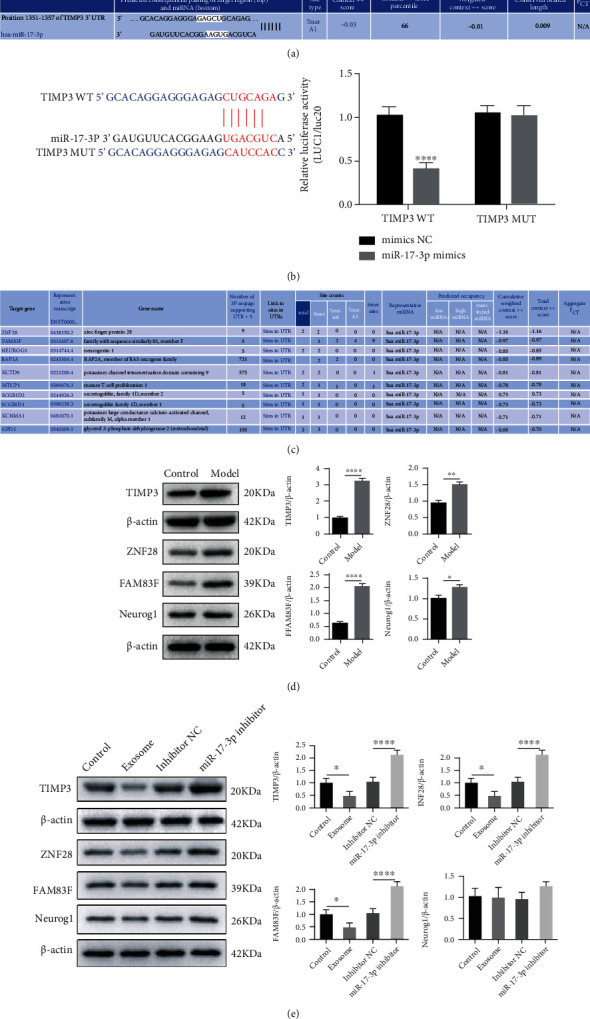
TIMP3 is a target of miR-17-3p. (a) The miR-17-3p binding site in the TIMP3 3′UTR was predicted using TargetScan7.2. (b) Dual-luciferase reporter assay was used to detect the luciferase activity cotransfected with 3′UTR of TIMP3 with the wild-type binding site or mutant binding site and miR-17-3p mimic. (c) The miR-17-3p targets were predicted with TargetScan7.2. (d) ZNF28, FAM83F, Neurog1, and TIMP3 levels in the cardiac I/R injury model were determined by Western blotting. Data are presented as the mean ± SD; ^∗∗^*P* < 0.01, model vs. control. (e) The effect of miR-17-3p on ZNF28, FAM83F, Neurog1, and TIMP3 levels was determined by Western blot analysis. The data are presented as the mean ± SD (*n* = 3); ^∗^*P* < 0.05; ^∗∗∗∗^*P* < 0.001.

We have demonstrated that miR-17-3p could regulate TIMP3 expression. Using TargetScan, we predicted the binding site for miR-17-3p in the 3′UTR of TIMP3 (Figure 3(a)). Dual-luciferase reporter gene assay showed that the luciferase activity was decreased in myocardium cells cotransfected with 3′UTR of TIMP3 plasmid containing a wild-type binding site and miR-17-3p mimic compared with the miR-17-3p mimic NC group (Figure 3(b)). To further confirm the importance of TIMP3, we predicted the targets of miR-17-3p using TargetScan. The results showed that ZNF28, FAM83F, and Neurog1 had the highest total context++ score (Figure 3(c)). Moreover, ZNF28, FAM83F, Neurog1, and TIMP3 expression levels were significantly elevated in the myocardial I/R injury mice compared to the control mice (Figure 3(d)). In addition, the exosomal miR-17-3p mimic and inhibitor significantly decreased and elevated ZNF28, FAM83F, and TIMP3 expression, in the cardiomyocytes, with TIMP3 having the highest fold changes in response to miR-17-3p modulation (Figure 3(e)). Taken together, exosomal miR-17-3p could negatively regulate the expression of its target genes, especially TIMP3.

### 3.4. TIMP3 Affected H_2_O_2_-Induced Programmed Necrosis of Cardiomyocytes Regulated by miR-17-3p In Vitro

**Figure 4:**
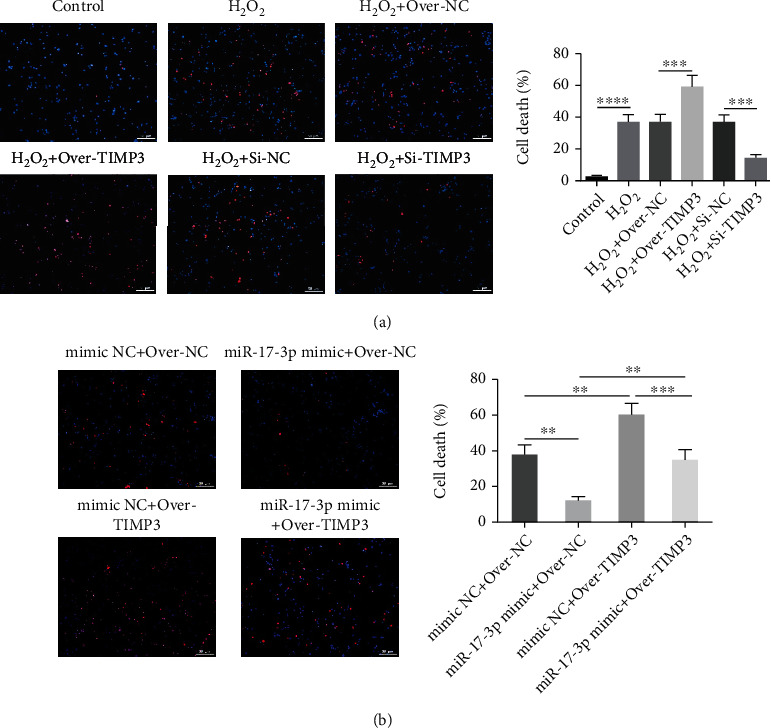
TIMP3 affected H_2_O_2_-induced programmed necrosis of primary cardiomyocytes and was regulated by miR-17-3p in vitro. (a) Necrotic cell death was assessed using PI staining of H9C2 cells following TIMP3 overexpression or knockdown. (b) The function of TIMP3 in necrotic cell death was identified using a rescue experiment. Representative images show PI-positive cells (bar = 100 *μ*m). Data are presented as the mean ± SD (*n* = 3); ^∗∗^*P* < 0.01; ^∗∗∗^*P* < 0.001; ^∗∗∗∗^*P* < 0.0001.

To understand the role of TIMP3 on programmed necrosis, the effect of TIMP3 overexpression and knockdown on H_2_O_2_-induced primary cardiomyocytes was investigated. We found that the number of primary cardiomyocytes undergoing programmed necrosis was dramatically increased following treatment with H_2_O_2_. This effect was aggravated by TIMP3 overexpression (Figure 4(a)). In contrast, programmed necrosis was greatly reduced by TIMP3 knockdown, suggesting that TIMP3 upregulation promoted H_2_O_2_-induced programmed necrosis in primary cardiomyocytes in vitro. To determine whether the function of TIMP3 in H_2_O_2_-induced programmed necrosis was affected by miR-17-3p, a rescue experiment was performed. The results demonstrated that the elevated number of necrotic cells observed in TIMP3-overexpressing cells could be partially reduced by treatment with miR-17-3p-expressing exosomes (Figure 4(b)). These results demonstrated that TIMP3 could increase H_2_O_2_-induced programmed necrosis of primary cardiomyocytes and this function could be regulated by miR-17-3p in vitro.

### 3.5. Exosomal miR-17-3p Greatly Alleviated Cardiac I/R Injury

**Figure 5:**
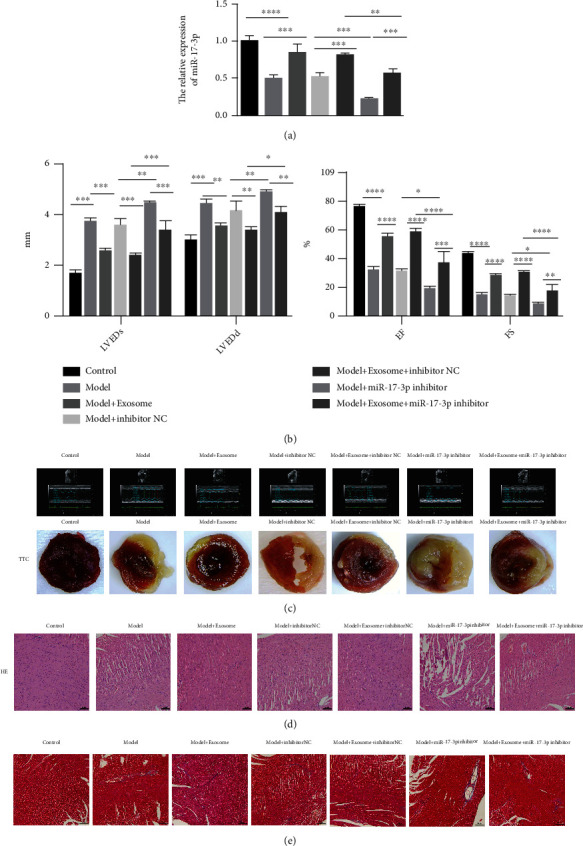
Exosomal miR-17-3p greatly alleviated cardiac I/R injury. (a) miR-17-3p expression was measured by qRT-PCR. (b) LVEDd, LVEDs, and EF of the left ventricle and fractional shortening (FS) at the left ventricular short axis after cardiac I/R injury were evaluated by echocardiography. (c) The effects of the exosomes and miR-17-3p inhibitor on the infarct size were determined by TTC staining.(d, e) The effect of exosomal miR-17-3p on inflammatory cell infiltration and fibrosis was investigated using H&E and Masson staining, respectively (bar = 100 *μ*m). Data are presented as the mean ± SD (*n* = 3); ^∗∗^*P* < 0.01; ^∗∗∗^*P* < 0.001; ^∗∗∗∗^*P* < 0.0001.

To further investigate the effects of exosomal miR-17-3p on cardiac I/R injury, a cardiac I/R injury mouse model was established. These mice were treated with exosomes or the miR-17-3p inhibitor. miR-17-3p expression was significantly increased following exosome treatment but decreased after treatment with the miR-17-3p inhibitor (Figure 5(a)). Echocardiography analysis showed that LVEDd and LVEDs were significantly elevated while EF (%) of the left ventricle and fractional shortening (FS) (%) at the left ventricular short axis were significantly decreased in the cardiac I/R injury model compared to the control group. However, treatment with exosomes resolved these changes. In contrast, treatment with the miR-17-3p inhibitor further aggravated these parameters in the cardiac I/R injury mice (Figure 5(b)). TTC staining showed that the infarct size was significantly increased in the cardiac I/R injury mice compared to the control mice. Consistent with these echocardiography results, the infarct size decreased following exosome treatment and further increased in the presence of the exosomal miR-17-3p inhibitor (Figure 5(c)). H&E and Masson staining indicated that inflammatory cell infiltration and fibrosis were increased in the injury model. However, these increases were dramatically reduced following exosome treatment. In contrast, treatment with the exosomal miR-17-3p inhibitor exacerbated inflammatory cell infiltration (Figures 5(d) and 5(e)).

**Figure 6:**
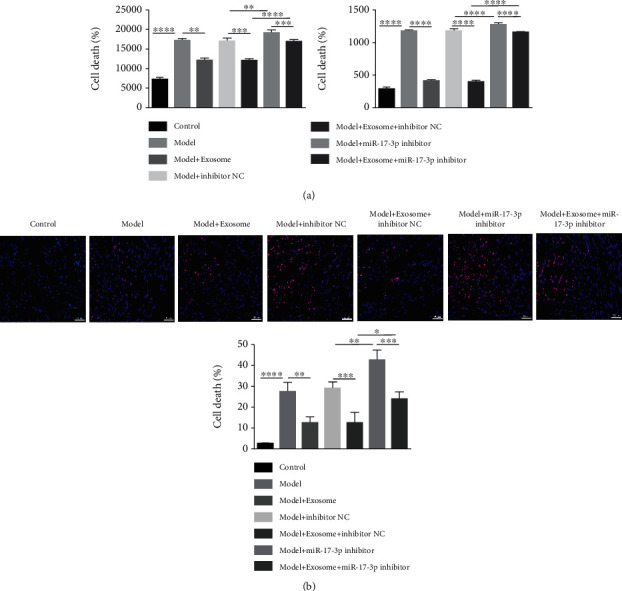
Exosomal miR-17-3p affected the LDH and TNF-*α* levels and necrotic cell death. (a) LDH and TNF-*α* levels were affected by exosomal miR-17-3p. (b) The effect of exosomal miR-17-3 on necrotic cell death was determined by PI staining (bar = 100 *μ*m). Data are presented as the mean ± SD (*n* = 3); ^∗∗^*P* < 0.01; ^∗∗∗^*P* < 0.001; ^∗∗∗∗^*P* < 0.0001.

To confirm these results, ventricular remodeling-related gene (*α*-MHC, *β*-MHC, ANP, and BNP) and fibrosis-related gene (*α*-SMA, Col3*α*1, and Col1*α*1) expression levels were measured by qRT-PCR. The results showed that *α*-SMA, Col1*α*1, Col3*α*1, ANP, BNP, and *β*-MHC expression levels were significantly elevated, while *α*-MHC expression levels were significantly decreased in the cardiac I/R injury model group compared with the control group. However, treatment with exosomes could partially rescue these expression changes. In contrast, the expression levels of *α*-SMA, Col1*α*1, Col3*α*1, ANP, BNP, and *β*-MHC were further increased, while *α*-MHC levels decreased after treatment with the exosomal miR-17-3P inhibitor (Supplementary Figure 1). Moreover, LDH and TNF-*α* levels were dramatically increased in the model group and further elevated following treatment with the exosomal miR-17-3p inhibitor. In contrast, the LDH and TNF-*α* levels significantly decreased following treatment with exosomes (Figure 6(a)). In addition, necrotic cells were significantly elevated in the model group compared to the control mice. Furthermore, the number of necrotic cells in model mice dramatically decreased following exosome treatment but they were further increased after treatment with the miR-17-3p inhibitor (Figure 6(b)). These results demonstrated that exosomal miR-17-3p greatly alleviated cardiac I/R injury.

## 4. Discussion

Myocardial I/R injury is a common pathophysiological process in ischemic heart disease and an important cause of aggravated myocardial injury. Programmed necrosis plays a crucial role in myocardial I/R injury. However, how exosomal miRNAs are regulated in programmed necrosis during myocardial I/R injury remains unclear. The present study demonstrated that exosomal miR-17-3p from peripheral blood alleviated cardiac I/R injury by inhibiting programmed necrosis via the miR-17-3p/TIMP3 axis.

Exosomes are nanoscale extracellular vesicles secreted by living cells and widely present in biological fluids. Exosomes carry proteins, lipids, and nucleic acids that can reflect the physiological and pathological conditions of the source cells. They play an important role in the exchange of material and information between cells [21]. An increasing number of studies have demonstrated that exosomal miRNAs could participate in myocardial I/R injury [21]. Exosomes or microvesicles from induced pluripotent stem cells deliver cardioprotective miRNAs and prevent cardiomyocyte apoptosis in the ischemic myocardium [22]. Plasma exosomes induced by remote ischemic preconditioning attenuate myocardial I/R injury by transferring with miR-24 [23]. In a previous study, miR-17-3p greatly contributed to exercise-induced cardiac growth and protected against myocardial I/R injury. However, the function of exosomal miR-17-3p in myocardial I/R injury remains unclear. In the present study, we found that miR-17-3p was significantly downregulated in peripheral blood exosomes from patients with cardiac I/R injury, suggesting that exosomal miR-17-3p might play an important role in myocardial I/R injury.

Multiple studies have demonstrated that programmed necrosis is involved in myocardial I/R injury [12]. Xu et al. [19] demonstrated that inhibition of mPTP opening regulates programmed necrosis and myocardial I/R injury. Sun et al. [24] showed that parkin regulates programmed necrosis and myocardial I/R injury by targeting cyclophilin-D. In addition, Wang et al. [20] found that the long noncoding RNA NRF could regulate programmed necrosis and myocardial injury during ischemia and reperfusion by targeting miR-873. MicroRNA-103 and microRNA-107 regulate programmed necrosis and myocardial I/R injury through targeting FADD [14]. These findings suggest that exosomal miRNAs can modulate myocardial I/R injury by regulating programmed necrosis. In the present study, we found that exosomal miR-17-3p attenuated H_2_O_2_-induced programmed necrosis of cardiomyocytes in vitro and exosomal miR-17-3p greatly alleviated cardiac I/R injury by inhibiting programmed necrosis in vivo.

miRNAs are involved in mRNA translation or degradation by binding the 3′UTR of mRNA, thereby achieving posttranscriptional gene regulation. Many miRNA targets are involved in cardiac I/R injury, including microRNA-193b/mastermind-like 1, miR-346/Bax, and miR-24-3p/RIPK1 [7, 25, 26]. In the present study, we found that TIMP3 was a direct target of miR-17-3p, consistent with a previous report [15]. Several studies have indicated that TIMP3 plays an important role in myocardial I/R injury. Specifically, Wang and Tong found that precondition of sevoflurane upregulates TIMP3 expression to alleviate myocardial I/R injury [27]. Purcell et al. showed that a matrix metalloproteinase-responsive hydrogel releasing TIMP-3 after myocardial infarction affected left ventricular remodeling [28]. Here, we demonstrated that exosomal miR-17-3p alleviated programmed necrosis during cardiac I/R injury by regulating TIMP3 expression. Liu et al. showed that TIMP3 overexpression protects against cardiac I/R injury by inhibiting myocardial apoptosis through the ROS/MAPK pathway [29]. Fuji et al. found that TIMP3 deficiency disrupts the hepatocyte E-cadherin/*β*-catenin complex and induces cell death in liver I/R injury [30]. These results suggested that TIMP3 might affect programmed necrosis in cardiac I/R injury by regulating ROS/MAPKs or cadherin/*β*-catenin pathways. However, the signaling pathway affected by TIMP3 needs to be further studied.

## 5. Conclusions

In summary, we demonstrated that miR-17-3p is significantly downregulated in peripheral blood exosomes from patients with cardiac I/R injury. The mechanism by which exosomal miR-17-3p alleviated programmed necrosis in cardiac I/R injury was through regulating TIMP3 expression. These findings could represent a potential treatment for I/R injury.

## Figures and Tables

**Table 1:** qRT-PCR primers.

Gene	Sequence (5′-3′)
U6-RT-F	CTCGCTTCGGCAGCACA
U6-RT-R	AACGCTTCACGAATTTGCGT
URP	CTCAACTGGTGTCGTGGA
hsa-miR-17-3p mature sequence	ACUGCAGUGAAGGCACUUGUAG
hsa-miR-17-3p reverse primer	CTCAACTGGTGTCGTGGAGTCGGCAATTCAGTTGAGCTACAAGT
rno-miR-17-1-3p mature sequence	ACUGCAGUGAAGGCACUUGUGG
rno-miR-17-3p reverse primer	CTCAACTGGTGTCGTGGAGTCGGCAATTCAGTTGAGCCACAAGT
miR-17-3p-F	ATAGACUGCAGUGAAGGC
a-SMA-mouse-F	CCCAACTGGGACCACATGG
a-SMA-mouse-R	TACATGCGGGGGACATTGAAG
Col3a1-mouse-F	CTGTAACATGGAAACTGGGGAAA
Col3a1-mouse-R	CCATAGCTGAACTGAAAACCACC
Col1a1-mouse-F	CTGGCGGTTCAGGTCCAAT
Col1a1-mouse-R	TTCCAGGCAATCCACGAGC
ANP-mouse-F	GCTTCCAGGCCATATTGGAG
ANP-mouse-R	GGGGGCATGACCTCATCTT
BNP-mouse-F	AGTCCTTCGGTCTCAAGGCA
BNP-mouse-R	CCGATCCGGTCTATCTTGTGC
*α*-mhc-mouse-F	TGAGTGGGAGTTTATCGACTTCG
*α*-mhc-mouse-R	CCTTGACATTGCGAGGCTTC
*β*-mhc-mouse-F	CCTGCGGAAGTCTGAGAAGG
*β*-mhc-mouse-R	CTCGGGACACGATCTTGGC
Actin-mouse-F	GTGACGTTGACATCCGTAAAGA
Actin-mouse-R	GCCGGACTCATCGTACTCC

## Data Availability

The data used to support the findings of this study are included within the article.

## Supplementary Materials

Supplementary Materials Supplementary Figure 1: exosomal miR-17-3p affected the expression of ventricular remodeling- and fibrosis-related genes. The expression levels of ventricular remodeling and fibrosis-related genes (e.g., *α*-SMA, Col3*α*1, Col1*α*1, *α*-MHC, *β*-MHC, ANP, and BNP) were measured by qRT-PCR using GAPDH as the control gene. Data are presented as the mean ± SD (*n* = 3); ^∗∗^*P* < 0.01; ^∗∗∗^*P* < 0.001; ^∗∗∗∗^*P* < 0.0001.
