# Supplementary material for: Exosomal miR-17-3p Alleviates Programmed Necrosis in Cardiac Ischemia/Reperfusion Injury by Regulating TIMP3 Expression
Source: Oxid Med Cell Longev. 2022 Jan 25;2022:2785113. doi: 10.1155/2022/2785113 (PMC8807034; doi:10.1155/2022/2785113)
Supplement: Supplementary Materials — Supplementary Figure 1: exosomal miR-17-3p affected the expression of ventricular remodeling- and fibrosis-related genes. The expression levels of ventricular remodeling and fibrosis-related genes (e.g., α-SMA, Col3α1, Col1α1, α-MHC, β-MHC, ANP, and BNP) were measured by qRT-PCR using GAPDH as the control gene. Data are presented as the mean ± SD (n = 3); ∗∗P < 0.01; ∗∗∗P < 0.001; ∗∗∗∗P < 0.0001. [file 2785113.f1.docx]

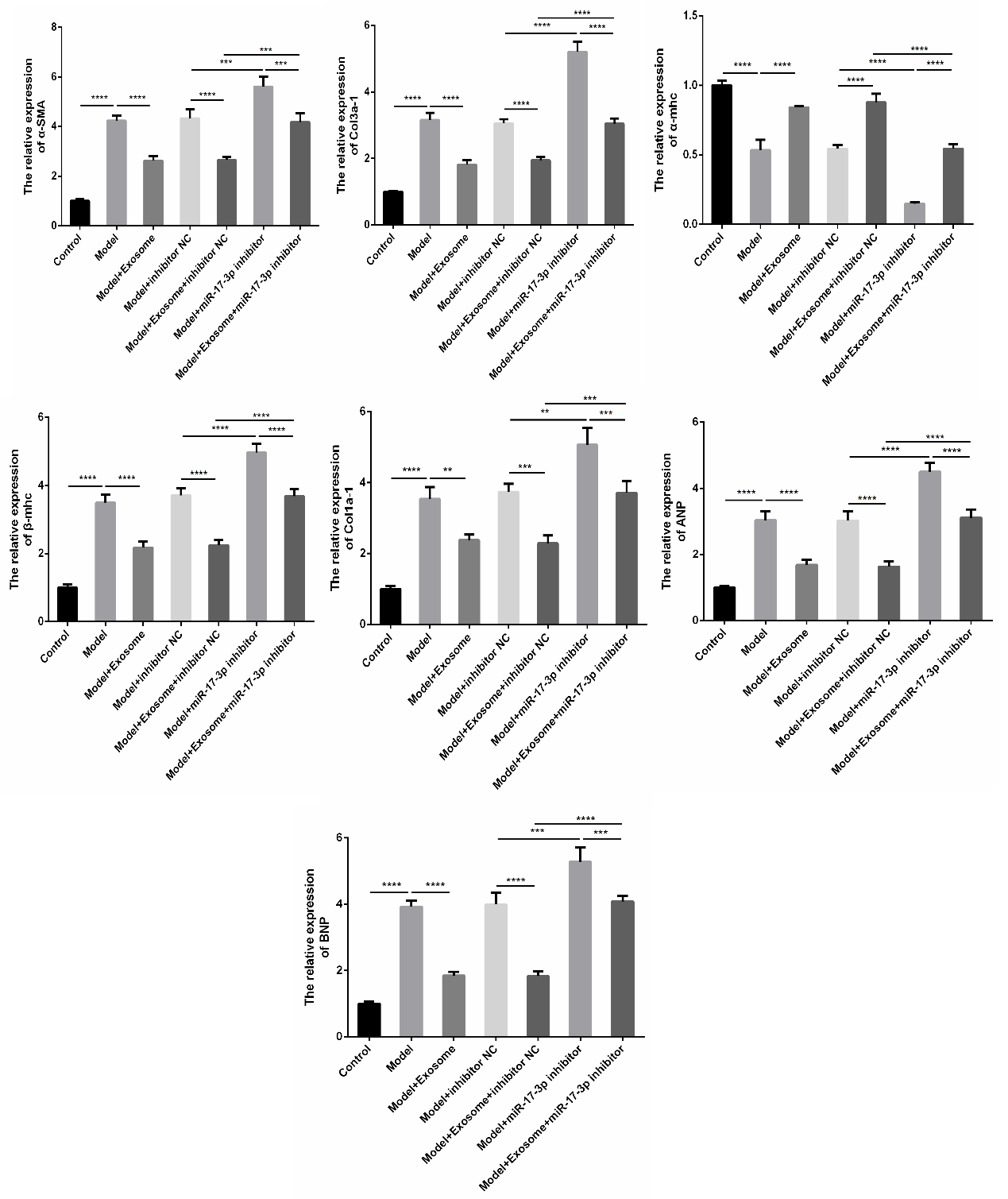


**Supplementary Figure 1. Exosomal miR-17-3p affected the expression of ventricular remodeling- and fibrosis-related genes.** The expression levels of ventricular remodeling and fibrosis-related genes (e.g., α-SMA, Col3α1, Col1α1, α-MHC, β-MHC, ANP, and BNP) were measured by qRT-PCR using GAPDH as the control gene. Data are presented as the mean ± SD (n = 3); **, P < 0.01; ***, P < 0.001; ****, P < 0.0001.
